# Mlo-Mediated Broad-Spectrum and Durable Resistance against Powdery Mildews and Its Current and Future Applications

**DOI:** 10.3390/plants13010138

**Published:** 2024-01-04

**Authors:** Antonín Dreiseitl

**Affiliations:** Department of Integrated Plant Protection, Agrotest Fyto, Ltd., 767 01 Kroměříž, Czech Republic; dreiseitl@vukrom.cz

**Keywords:** barley, *Hordeum vulgare*, powdery mildew, *Blumeria hordei*, durable resistance, Mlo resistance, resistance gene postulation, virulence frequency

## Abstract

Mlo is a well-known broad-spectrum recessively inherited monogenic durable resistance to powdery mildew caused by *Blumeria hordei* found first in barley, originally in an induced mutant in 1942 and later in other mutants and also in Ethiopian landraces. The first commercial varieties possessing Mlo resistance were released during 1979–1986, but these often showed symptoms of necrotic leaf spotting associated with reduced grain yield. However, this yield penalty was successfully reduced by breeding Mlo-resistant varieties of spring barley predominate in Europe; for example, in the Czech Republic, their ratio surpassed 90% of the total number of newly released varieties. However, outside Europe, Mlo-varieties are not yet popular and can be exploited more widely. Winter barley varieties are generally non-resistant, but the use of Mlo for their breeding is controversial despite the limited adaptability of the pathogen to this resistance. The renewal of mechanically disturbed epidermal plant cell walls, including the penetration of mildews, is common in plants, and the Mlo-type resistance is exploited in many other crop species, including wheat.

## 1. Introduction

Broad-spectrum resistance is a general term that involves different kinds of resistance to plant pathogens, including specific resistances via the accumulation of QTLs in adult plants, and non-specific or non-host resistance that confers resistance to several diseases [1,2,3,4,5]. In the case of barley powdery mildew, due to its remarkable ability to quickly overcome plant immunity [6], the durability of resistance based on non-specificity is a key requirement.

Non-specific resistance simply means that no virulent pathotype exists, and to confirm that this requires testing the resistant host against a wide range of pathogen isolates or populations. There is a high probability that virulent pathotypes to a new and effective resistance exist in the geographical region where the source accession(s) is found and where the pathogen has already had a chance to adapt to the resistance. In addition to this, two centers of virulence diversity and complexity of *Blumeria hordei*, M. Liu and Hambl. (*Bh*), that cause powdery mildew (PM) are found in barley (*Hordeum vulgare* L.). The first is located in the center of wild barley diversity (*H*. *v*. subsp. *spontaneum*), where the host and the pathogen have coexisted for a long time. It is characterized by a broad spectrum of virulences against resistance genes that are present in this barley subspecies [7]. The second center has developed recently in Europe [8], especially in the central and northwest areas, where there has been a massive exploitation of specific resistances in breeding crop varieties [9,10].

Specific resistances are often initially effective against known pathotypes (virulence frequency—VF = 0%). For example, no virulent isolates of the three corresponding *Ml* resistance genes (*aLv*, *p,* and *Ve*) were recorded before 2009–2012 (Table 1). This may also be the case of Roxana (Ro) resistance [11], but the inclusion of ‘Ro-varieties’ in a differential set only took place in 2008. This was some time after the varieties with this resistance had already been grown in Germany, and hence a small proportion of virulent isolates found in 2008 [12] were probably pathotypes originating in that country.

Increasing VF in the pathogen population is followed by decreased varietal resistance in the field (Table 2). There are many examples of this phenomenon, and one of the best known is the case of varieties with *Mla13* that maintained their resistance in the Czech Republic for ten years (1976–1985). The resistance was subsequently overcome there and throughout Europe shortly afterward [21]. Since 1989, varieties possessing *Mla13* have been among the most susceptible [22].

After performing tests against many isolates and multiple locations, only resistances based on *mlo* and *Mlp* genes remained effective, and both were considered potentially non-specific [24]. However, in the samples of the *Bh* population collected from wild barley in Israel, VF to *Mlp* (VF*p*) was 96% in 1997 and 100% in 1999 [7]. In the Czech population (central Europe), virulence against *Mlp* was not found before 2012 [17], but after varieties carrying this gene started to grow, VF*p* reached almost 70% in 2023 [19].

When 1 383 accessions of the U.S.A. wild barley collection were tested with mostly European *Bh* isolates, it was found that 123 accessions were resistant to all of them [25]. These accessions were tested with 38 Israeli isolates, and apart from the check lines containing *mlo* and *Mlhb2*, only PI 466634 remained resistant [26]. Therefore, the derivatives from bulbous barley (*Hordeum bulbosum* L.) [27,28] and wild barley accession PI 466634 may be potential sources of non-specific resistance.

## 2. Mlo Resistance in Barley

A broad-spectrum recessively inherited monogenic resistance to powdery mildew was first recognized in an induced mutant M66 derived from Haisa [29]. The same resistance was subsequently found in other induced barley mutants and landraces collected in Ethiopia [30]. These genes were assigned to a new locus designated Mlo [31]. Ten alleles in the mutants and the alleles present in the landraces were numbered *mlo1*–*mlo10* and *mlo11*, respectively [32], and were located at this locus, which has at least three sites [33] on the barley chromosome 4HL [34]. Many other mutants with different *mlo* alleles have since been reported. Mutations with a similar phenotype, characterized by the occurrence of occasional small mildew colonies and an identical resistance function, can occur at these locus sites. The number of colonies may vary according to environmental conditions and the presence of an *mlo* allele and the genotype of the recipient variety, which may contain specific resistance genes [35].

Over a period of four years, the first three commercial spring barley varieties carrying *mlo11*, derived from potentially different Ethiopian landraces, were registered (Atem in 1979, Salome in 1981, and Apex in 1982). These were followed by the registration of the first variety (Alexis 1986) with an induced Mlo resistance (*mlo9*) [35] derived from a Czech EMS mutant SZ 5139 [36]. This mutant was obtained from a well-known domestic semi-dwarf and highly tillering variety Diamant—an Rtg mutant from Valticky, a world donor of high malting quality grain.

In the Czech Republic, spring barley is an important crop used mostly for malt and beer production, and both these commodities are exported. However, the cultivated area of the crop fell from a yearly average of 624,000 hectares during 1975–1982 to 220,000 ha during 2016–2021. Despite this reduction, the country is still a good seed market for Central and Western European companies. This explains why 164 commercial varieties bred in numerous companies and breeding stations in nine countries were registered here over the last 31 years (Table 3). This set of varieties truly reflects the focus of the breeding programs in these European regions.

In the inland Czech Republic, PM is the most frequent disease of non-resistant barley varieties [39], and therefore, the immigration of airborne spores with the potential to be transmitted for hundreds of kilometers [40] is not limited. Gene flow is an important evolutionary force [41] and, together with a suite of other factors [42], PM has higher importance than elsewhere, and the demand for resistant varieties has always been important.

Varieties with Mlo resistance have been tested in the country since 1986, initially only in registration trials. The first commercial variety with a broad-spectrum durable Mlo resistance was the Czech-bred Forum, which was registered in 1993 [43]. At the end of 2023, 114 of these varieties were registered in the Czech Republic, and 92 of these varieties registered up to 2020 were published (Table 4).

Varieties carrying Mlo attained almost 96% of newly registered strains in the last monitored period (Table 5), and for 38 years, they were the most resistant to PM [22].

Genotypes carrying induced as well as naturally occurring *mlo* alleles were originally associated with negative characteristics, such as necrotic leaf spotting and reduced grain yield. But even in the early 1990s when many fewer Mlo commercial varieties existed, it was reported that these negative attributes “have been overcome by recent breeding work” and “absence of necrotic leaf spotting may be an easy selection criterion for removing undesirable pleiotropic effects of the *mlo* resistance genes” [35]. Hundreds of commercial Mlo varieties that were released in Europe have performed well in trials of breeding companies and registration trials, and they are widely accepted on the seed market and among growers. In some specific conditions and on some varieties, more colonies of the pathogen and more necrotic leaf spotting can occur, but there are no obstacles to the further use of this admirable non-specific durable resistance because the benefits of strong Mlo-based resistance outweigh any small penalties [47,48].

There are two barley crops grown concurrently in much of Europe: spring, whose PM resistance is generally high and mostly based on Mlo, and winter, where varieties are often susceptible despite the frequent presence of more than one gene of specific resistance. Therefore, there is a question regarding the use of Mlo resistance in winter barley. The answer requires the knowledge of whether there is a potential for the pathogen to adapt to such “special” resistance response. To investigate this, a barley *mlo* mutant was inoculated in 37 vegetative reproduction cycles with a pure field *Bh* isolate and its progeny. The results showed that the number of colonies and the number of spores per colony increased, leading to a significantly higher number of spores per leaf area compared with a variant when the mutant was inoculated with the original field isolate [49]. When varieties with Mlo were already widely grown, subsequent tests with field isolates at several locations showed that the pathogen could adapt to Mlo to a limited extent [50].

Based on the available data, it was predicted that Mlo will be a very durable resistance. Nevertheless, if Mlo-resistant spring and winter barley varieties are grown extensively and their areas overlap, it is possible that the powdery mildew fungus will slowly evolve with increased aggressiveness (partial virulence) and gradually cause disease that may approach the threshold level for crop losses [35]. This conclusion is generally accepted, and Mlo resistance has not been used in the breeding of commercial winter barley varieties.

Despite this reservation, there were two attempts to use Mlo in winter barley breeding programs. The Czech winter barley KM 2099 was tested in domestic registration trials in 1990–1993 and exhibited some favorable agronomic traits, including effective PM resistance based on an *mlo* allele [51]. Because it was not morphologically uniform, reselection of this variety was considered. However, the presence of Mlo resistance did not support further testing of KM 2099 or its selected lines, and similar breeding efforts were curtailed. The second example relates to a Polish research project that started in 2005 and aimed at the transfer of Mlo into winter barley genotypes. A set of lines with the possible presence of Mlo resistance and acceptable agronomical traits were bred, and one line (BKH 5735) was selected [52]. The fate of this variety is not known.

The protection of European spring barley against PM is based mainly on Mlo resistance, but the reported results do not support the use of Mlo resistance for winter barley breeding under present European conditions where vegetative reproduction of the pathogen could occur throughout the year in winter and spring barleys carrying Mlo. Mlo does not only provide resistance against PM but its action is manifested by a general renewal of mechanical damage of epidermal plant cell walls (including penetration of PM), although possible adaptation to partial virulence is known. Therefore, without more research on this topic, there must be caution regarding the breeding of winter barley carrying Mlo until the following unanswered question can be addressed—“Spring and winter barley cultivars with the *mlo* resistance gene to powdery mildew—is there a threat of pathogen adaptation?” [51].

## 3. Mlo Resistance in Other Crops and Plant Species

In plants with Mlo resistance, the renewal of mechanically disturbed epidermal plant cell walls is wound-sealed by the formation of a callose-rich cell wall apposition below the encounter site. This fundamental mechanism is generally found in higher plants, and it was predicted that such kind of mildew resistance should also occur in other plant species [35]. This prediction inspired the search for Mlo resistance in a wide range of plants, and until 2017, such functionally validated resistance was detected in 12 other species in addition to barley [53]. Based on continuing research, the number and range of plant species with Mlo resistance are increasing [54,55,56,57], and wheat, for example, could result in the use of Mlo resistance without pleiotropic effects causing growth penalties [58,59]. New technologies, including CRISPR-Cas9, can further accelerate the exploitation of Mlo resistance for the protection of many crops [60].

## 4. Conclusions

- Mlo resistance in barley is a very effective broad-spectrum durable resistance against powdery mildew based on the recessive *mlo* gene.
- The yield penalty for Mlo resistance, known from research conducted several decades ago, was successfully reduced by breeding.
- Outside Europe, using Mlo in barley breeding is not a high priority and has great potential for the increased utilization of this resistance.
- Even though the pathogen has a limited ability to adapt, the joint use of Mlo in both spring and winter barleys could be risky in areas where these crops are grown extensively.
- The renewal of mechanically disturbed epidermal plant cell walls, including the penetration of mildews, is common in plants, and Mlo-type resistance is found in many crops.
- The detection of this resistance type and related research probably continue in other plant species.

## Figures and Tables

**Table 1:** Virulence frequency (VF) in the Czech *Blumeria hordei* population that overcomes specific powdery mildew (*Ml*) resistance genes in barley varieties.

Variety	Registration	*Ml* Gene	Year	VF (%)	Year	VF (%)	Year	VF (%)
Kangoo ^1^	2008	*Ro*			2008	2.4 ^2^	2014	71.7 ^3^
Laverda ^4^	2007	*aLv*	2008	0 ^5^	2009	0.7 ^6^	2011	23.3 ^3^
Saturn ^7^	2012	*p*	2011	0 ^6^	2012	0.7 ^6^	2019	69.5 ^8^
SU Celly ^9^	2020	*Ve*	2009	0 ^6^	2011	0.7 ^6^	2023	26.2 ^8^

^1^ [13], ^2^ [12], ^3^ [14], ^4^ [15], ^5^ [16], ^6^ [17], ^7^ [18], ^8^ [19], ^9^ [20].

**Table 2:** The breakdown of specific powdery mildew (*Ml*) resistance genes present in barley varieties recorded in Czech registration trials.

*Ml* Gene(s)	Variety	Registration	Average Resistance ^1^			
*a6*	Ametyst ^2^	1972	1971	7.20 ^3^	1977	4.33
*a6*, *g*	Rapid ^2^	1976	1974	4.93	1977	**3.67** ^4^
*a7*, *g*	Elgina ^5^	1973	1971	**8.90**	1974	7.14
*a7*	Diabas ^2^	1977	1975	5.29	1978	**4.33**
*a9*	Spartan ^2^	1977	1976	8.60	1983	**3.38**
*a12*	Zefir ^2^	1981	1978	7.00	1981	**3.24**
*a13*, *g*	Koral ^2^	1978	1977	**9.00**	1986	5.50
*a13*, *g*	Krystal ^2^	1981	1984	**9.00**	1989	**3.95**

^1^ [22]; ^2^ [23]; ^3^ 9 = resistant; ^4^ Bold—the most resistant/susceptible variety in that year; ^5^ [9].

**Table 3:** Spring barley varieties registered in the Czech Republic during 1993–2023: numbers of those with Mlo powdery mildew resistance and their country and region of origin.

Country of Origin	Region of Europe	Total	Mlo	%
Germany	Central ^1^	57	40	70.2
Czech Republic	Central	35	22	62.9
France	Northwest ^2^	34	31	91.2
Netherlands	Northwest	11	3	27.3
Denmark	Northwest	9	7	77.8
Slovakia	Central	6	2	33.3
United Kingdom	Northwest	6	6	100.0
Austria	Central	3	0	0.0
Switzerland	Central	3	3	100.0
Sum		164	114	

^1^ Region of Europe [37], ^2^ Subregion of Europe [38].

**Table 4:** List of 92 spring barley varieties registered in the Czech Republic during 1993–2020 and possessing Mlo resistance against powdery mildew.

Variety	Country	Registration	Reference(s)	Variety	Country	Registration	Reference(s)
	of Origin ^1^				of Origin ^1^		
Accordine	G	2018	[20]	LG Monus	F	2017	[20]
Acrobat	F	2008	[13,44]	LG Nabuco	F	2018	[20]
Adam	G	2020	[19,20]	LG Tosca	F	2020	[20]
Advent	CZ	2009	[13]	Libuše	G	2016	[20]
AF Cesar	CZ	2014	[45]	Madeira	G	1999	[13,44]
Aksamit	CZ	2007	[13]	Madonna ^3^	G	1998	[44]
Aktiv	CZ	2008	[13]	Manta	G	2016	[20]
Aligator	G	2016	[20]	Marthe ^2^	G	2008	[44]
Atribut	CZ	1996	[23]	Monalisa	F	2011	[45]
Avus	G	2020	[20]	Montoya	G	2014	[45]
Berlioz	F	2010	[13]	Nitran	SK	2004	[46]
Bernstein	F	2008	[13,44]	Nordus	G	1998	[13,44]
Biatlon	UK	2003	[44]	Odyssey	F	2014	[45]
Blanik ^2^	NL	2007	[44]	Olbram	CZ	1996	[23]
Bojos	CZ	2005	[13]	Olympic	F	2013	[45]
Braemar	UK	2006	[44]	Ovation	F	2017	[20]
Britney	G	2014	[45]	Overture	F	2014	[45]
Calgary	F	2003	[13,44]	Paulis ^3^	CZ	2010	[13]
Class (Topic)	F	2005	[13,44]	Petrus	F	2013	[45]
Concerto	F	2011	[45]	Philadelphia	G	2002	[13,44]
Cosmopolitan	DK	2019	[20]	Pilote	CH	2018	[20]
Danielle	G	2013	[45]	Poet	DK	2007	[13,44]
Delphi	DK	2011	[45]	Prestige	F	2002	[13,44]
Despina	G	2011	[45]	Prunella	F	2015	[45]
Eurojet	G	2004	[13]	Publican	UK	2008	[13,44]
Fandaga	G	2020	[20]	Radegast	CZ	2005	[13]
Forman	G	2017	[20]	Respekt ^3^	CZ	2003	[13]
Forum	CZ	1993	[23]	RGT Otakar	F	2014	[45]
Francin ^3^	CZ	2014	[45]	Runner	G	2019	[20]
Gladys	NL	2010	[13]	Sabel	UK	2001	[13]
Henley	F	2009	[13]	Saloon	UK	2002	[44]
Heris	CZ	1998	[23]	Sanette	CH	2015	[45]
Ismena	G	2019	[20]	Shuffle	G	2013	[45]
Jersey	NL	2000	[44]	Signora	F	2009	[46]
Kontiki	DK	2009	[13]	Signum	CZ	2012	[45]
Krona	G	1996	[13]	Sladar	SK	2010	[46]
Kvorning	G	2015	[45]	Solist	G	2015	[45]
KWSAmadora	G	2015	[45]	Soulmate	DK	2017	[20]
KWS Asta	G	2014	[45]	Spitfire ^3^	CZ	2018	[20]
KWS Fantex	G	2018	[20]	Streif	G	2009	[13]
KWS Irina	G	2014	[45]	SU Zaza	G	2014	[45]
Laudis 550	CZ	2013	[45]	Tango	F	2016	[20]
Laureate	G	2019	[20]	Westminster	UK	2007	[13,44]
Leenke	G	2017	[19,20]	Wiebke	G	2012	[45]
LG Aurus	F	2019	[20]	Xanadu ^2^	G	2006	[44]
LG Ester	F	2020	[20]	Zhana	F	2013	[45]

^1^ CZ—Czech Republic, DK—Denmark, F—France, G—Germany, NL—Netherlands, SK—Slovakia, CH—Switzerland, UK—United Kingdom. ^2^ In the cited article, Blanik, Marthe, and Xanadu are given the code numbers Ceb 0367, NORD 02/2338, and NORD 00/2310, respectively. ^3^ Heterogeneous variety, beside Mlo resistance, a line with specific resistance gene(s) is present.

**Table 5:** Number of spring barley varieties newly registered in the Czech Republic over a period of 31 years and the numbers and proportion of those carrying the Mlo resistance.

Years	Total	Mlo	%
1993–1995	8	1	12.5
1996–2000	17	8	47.1
2001–2005	21	12	57.1
2006–2010	31	20	64.5
2011–2015	35	27	77.1
2016–2020	29	24	82.8
2021–2023	23	22	95.7
Sum	164	114	

## Data Availability

All relevant data are presented in the article.
